# Whole-Genome Sequencing-Based Resistome Analysis of Nosocomial Multidrug-Resistant Non-Fermenting Gram-Negative Pathogens from the Balkans

**DOI:** 10.3390/microorganisms11030651

**Published:** 2023-03-03

**Authors:** Slavil Peykov, Tanya Strateva

**Affiliations:** 1 Department of Genetics, Faculty of Biology, Sofia University “St. Kliment Ohridski”, 8, Dragan Tzankov Blvd., 1164 Sofia, Bulgaria; 2 Department of Medical Microbiology, Faculty of Medicine, Medical University of Sofia, 2, Zdrave Str., 1431 Sofia, Bulgaria; 3 BioInfoTech Laboratory, Sofia Tech Park, 111, Tsarigradsko Shosse Blvd., 1784 Sofia, Bulgaria

**Keywords:** non-fermenting gram-negative bacilli, Balkans, whole-genome sequencing, multidrug resistance (MDR), extensive drug resistance (XDR), resistome analysis

## Abstract

Non-fermenting Gram-negative bacilli (NFGNB), such as *Pseudomonas aeruginosa* and *Acinetobacter baumannii*, are among the major opportunistic pathogens involved in the global antibiotic resistance epidemic. They are designated as urgent/serious threats by the Centers for Disease Control and Prevention and are part of the World Health Organization’s list of critical priority pathogens. Also, *Stenotrophomonas maltophilia* is increasingly recognized as an emerging cause for healthcare-associated infections in intensive care units, life-threatening diseases in immunocompromised patients, and severe pulmonary infections in cystic fibrosis and COVID-19 individuals. The last annual report of the ECDC showed drastic differences in the proportions of NFGNB with resistance towards key antibiotics in different European Union/European Economic Area countries. The data for the Balkans are of particular concern, indicating more than 80% and 30% of invasive *Acinetobacter* spp. and *P. aeruginosa* isolates, respectively, to be carbapenem-resistant. Moreover, multidrug-resistant and extensively drug-resistant *S. maltophilia* from the region have been recently reported. The current situation in the Balkans includes a migrant crisis and reshaping of the Schengen Area border. This results in collision of diverse human populations subjected to different protocols for antimicrobial stewardship and infection control. The present review article summarizes the findings of whole-genome sequencing-based resistome analyses of nosocomial multidrug-resistant NFGNBs in the Balkan countries.

## 1. Introduction

Non-fermenting Gram-negative bacilli (NFGNB) are a heterogeneous group of aerobic, non-spore-forming bacteria that do not utilize carbohydrates via fermentation as an energy source [1]. Some species, such as *Pesudomonas aeruginosa*, *Acinetobacter baumannii*, and *Stenotrophomonas maltophilia*, have already been recognized as important nosocomial pathogens with significant contributions to mortality in hospitals worldwide [2]. Moreover, the first two species mentioned are part of the ESKAPE (*Enterococcus faecium*, *Staphylococcus aureus*, *Klebsiella pneumoniae*, *A. baumannii*, *P. aeruginosa*, and *Enterobacter* species) pathogens that represent a global therapeutic problem worldwide [3]. They frequently “escape” from the most commonly used antimicrobial treatment via the acquisition and/or development of multiple resistance mechanisms [4]. Due to the potential resistance to a high number of antibiotics, *P. aeruginosa* and *A. baumannii* are designated as urgent/serious threats by the Centers for Disease Control and Prevention and are included in the World Health Organization’s (WHO) list of critical priority pathogens [5,6,7,8]. Particularly problematic are carbapenem-resistant *P. aeruginosa* (CRPA) and *A. baumannii* (CRAB), most of which are simultaneously multidrug-resistant (MDR, non-susceptible to at least one agent in more than three antimicrobial categories) or extensively drug-resistant (XDR, non-susceptible to at least one agent in all but two or fewer antimicrobial categories) [4,9]. Treating the infections caused by them represents a serious challenge for clinicians, and often novel generation antibiotics, such as ceftolozane/tazobactam, ceftazidime/avibactam, and cefiderocol, should be applied [10,11,12]. In other occasions, the treatment requires even the reconsideration of old antimicrobial drugs, such as polymyxins, which were considered too toxic for clinical use before [13]. One of the two commercially available polymyxins, colistin, is now considered as a last-line therapy for infections caused by XDR NFGNB from the ESKAPE group [14].

The most common and severe NFGNB is *P. aeruginosa*, which is frequently found to be involved in a wide variety of nosocomial infections, particularly infecting patients with predisposing factors, such as burn victims, immunocompromised hosts, or those with metabolic disorders [15,16,17]. It is also recognized as a predominant cause of pulmonary disease and mortality in patients with cystic fibrosis [18].

Furthermore, recent reports highlighted that up to 50% of neutropenic patients diagnosed with an infection due to MDR *P. aeruginosa* isolates also received inappropriate antibiotic empirical therapy, and this was associated with higher mortality rates [19]. In total, from all estimated 541,000 deaths (95% uncertainty intervals: 370,000–763,000) associated with bacterial antimicrobial resistance (AMR) in the WHO European region in 2019, 43,801 were caused by *P. aeruginosa* and 29,000 of the causative agents were CRPA [20].

Despite that *A. baumannii* is not considered as a highly virulent pathogen, it is found to be responsible for many cases of ventilator-associated pneumonia, bloodstream infections, urinary tract infections, and meningitis in critically ill patients [21]. It is also considered as a serious burden for many hospital settings globally mainly due to its immense ability to acquire and/or upregulate AMR genetic determinants [22]. It is worth noting that a retrospective study of US military personal wounded in Iraq or Afghanistan highlighted *A. baumanniii* as the most commonly isolated pathogen from open tibial fractures [23]. Moreover, the percentage of MDR isolates recovered from patients in a military medical center in the US was 2.5 times higher among war casualties deployed overseas (52%) than among local patients (20%) [24]. This statistic is of a particular concern in the light of the ongoing conflict in Ukraine with an estimated number of up to 200,000 military casualties on all sides. In the WHO European region, *A. baumannii* was one of the seven pathogens responsible for 27,206 deaths associated with AMR in 2019 and 18,100 of these isolates were CRAB [20].

*S. maltophilia* is increasingly recognized as an emerging cause for healthcare-associated infections (HAIs) in intensive care unit (ICU) patients, life-threatening diseases in immunocompromised patients, and severe pulmonary infections in individuals with cystic fibrosis [25,26]. It ranks third among NFGNB (after *P. aeruginosa* and *Acinetobacter* species) as a cause of HAIs [27,28]. Moreover, a recent study focused on respiratory co-infections in COVID-19 patients in an ICU in China, which determined *S. maltophilia* to be the most frequently isolated pathogen in the severe patients group and in the critical patients group [29]. These findings emphasize the importance of this pathogen for the Balkan countries since four of them are on the list for highest number of COVID-19 deaths worldwide per one million population [30]. *S. maltophilia* possesses an intrinsic resistance towards β-lactams, including carbapenems, due to the production of two β-lactamases (L1 metallo-β-lactamase (MBL) and L2 inducible cephalosporinase) [31]; therefore, the use of this class of antibiotic is not considered as a treatment option [32,33]. The last resort antibiotic colistin is also not suitable despite the demonstrated in vitro activity because half of the isolates are actually resistant to this antibiotic [34]. In addition, a recent study described a colistin-degrading protease in an environmental *S. maltophilia* isolate [35]. Co-cultivation experiments demonstrated that this enzyme can inactivate colistin and thereby protect an otherwise susceptible *P. aeruginosa* strain. Similar enzyme protection may seriously affect the treatment options for patients co-infected with *S. maltophilia* and other NFGNBs.

Antimicrobial resistance surveillance and outbreak investigations are mandatory to restrain NFGNB-caused infections in hospital wards, especially ICUs. Analysis of the AMR in the WHO European region in 2019 showed drastic differences in the proportions of NFGNB with resistance towards key antibiotics in different European Union/European Economic Area countries [20]. The number of resistant isolates trend to increase following the axes “west–east” and “north–south” highlighting the Balkans as a high priority region. There is no universal agreement on the components of the easternmost of Europe’s southern peninsulas [36].

## 2. Antimicrobial Resistance in NFGNBs in the Balkan States

In this review article we summarize the whole-genome sequencing-based resistome analyses of isolates from the Balkan countries (Albania, Bosnia and Herzegovina, Bulgaria, Croatia, Greece, Kosovo, Montenegro, North Macedonia, Romania, Serbia, and Turkey) found in the literature. The entire Balkan region is characterized by a high degree of ethnic diversity and fragmentation. Despite that, it is surrounded by seas, and the peninsula is not cut off from neighboring regions to the east, west, or south. This creates a lot of crossroads for traffic passing to and from Anatolia, the Italian peninsula, and the eastern Mediterranean region. Moreover, the Balkan countries are very diverse in aspects, such as European Union (EU) membership (Bulgaria, Croatia, Greece, and Romania) or Schengen Area membership (Croatia and Greece), all of which affects the AMR monitoring on different levels. The current situation in the Balkans includes a migrant crisis and reshaping of the Schengen Area border. This results in the collision of diverse human populations subjected to different protocols for antimicrobial stewardship and infection control. All these circumstances together contribute to the wide spread of MDR pathogens, including MDR-NFGNBs, on the Balkans.

**Figure 1:**
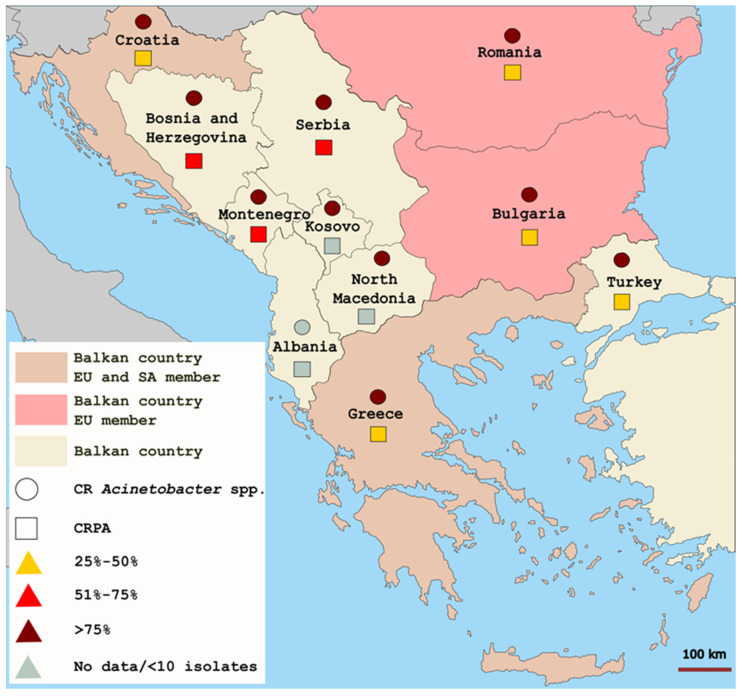
Geographic distribution of invasive carbapenem-resistant *Acinetobacter* spp. and *P. aeruginosa* on the Balkans according to the 2020 annual report of the ECDC [37]. CR, carbapenem-resistant; CRPA, carbapenem-resistant *P. aeruginosa*; EU, European Union; SA, Schengen Area.

According to the 2020 annual report of the European Centre for Disease Prevention and Control (ECDC), the prevalence of invasive CRPA isolates on the Balkans exceeded 30% with the highest values reported in Montenegro (72.7%) and Serbia (65.9%) and the lowest in Croatia (30.3%), Greece (35.7%), and Turkey (36.2%) [37]. The corresponding frequency values for invasive carbapenem-resistant *Acinetobacter* spp. isolates were the highest in Montenegro (100%) and Bosnia and Herzegovina (97.9%), and the lowest were in Bulgaria (82.9%) and Kosovo (84.7%). These numbers highlight the Balkan countries as a reservoir for CRPA and CRAB isolates in Europe [37]. The geographic distribution of carbapenem-resistant *Acinetobacter* spp. and *P. aeruginosa* is shown in Figure 1.

**Figure 2:**
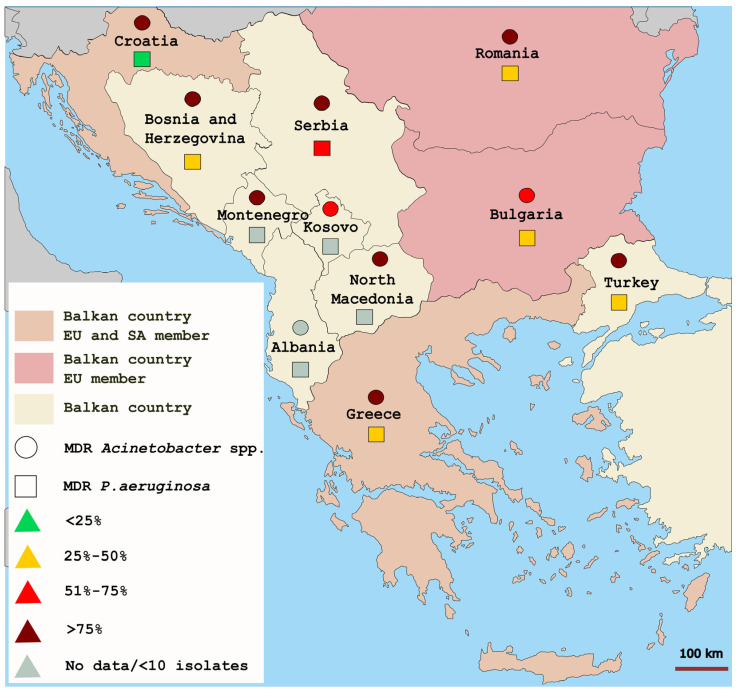
Geographic distribution of invasive multidrug-resistant *Acinetobacter* spp. and *P. aeruginosa* on the Balkans according to the 2020 annual report of the ECDC [37]. MDR, multidrug-resistant; EU, European Union; SA, Schengen Area.

On the other hand, the prevalence of MDR (combined resistance to carbapenems, fluoroquinolones, and aminoglycosides) *Acinetobacter* spp. exceeded 70%, with the highest values reported in Serbia (95.9%) and Croatia (95.1%) and the lowest in Kosovo (71.2%) and Bulgaria (72.9%). The incidence of MDR (combined resistance to ≥3 antimicrobial groups, including piperacillin-tazobactam, ceftazidime, carbapenems, fluoroquinolones, and aminoglycosides) *P. aeruginosa* varied widely, from 11.6% (Croatia) to 61.4% (Serbia) [37]. The report lacks data for Albania. The data are presented in Figure 2.

Monitoring these trends requires the adaptation of new technologies, such as whole-genome sequencing (WGS), as a tool to precisely determine the AMR mechanisms. It provides a vast amount of information and the highest possible resolution for pathogen subtyping. Moreover, the generated sequencing data by AMR surveillance programs, such as the Global Antimicrobial Resistance Surveillance System (GLASS) implemented by WHO, may guide the development of rapid and sensitive diagnostic tools addressing the global antibiotic resistance epidemic [38].

The next section presents a brief introduction into the used sequencing technologies with their strengths and weaknesses for resistome analysis of clinical NFGNB isolates.

## 3. Whole-Genome Sequencing of Bacterial Pathogens

WGS has been globally adopted as a tool of choice for resistome studies in the field of clinical microbiology [39]. The first sequenced genome of a self-replicating, free-living bacterium was that of *Haemophilus influenzae Rd* and its analysis identified several genetic determinants involved in antibiotic resistance [40]. Many other bacterial genomes have been sequenced in the next several years including the genomes of the NFGNB, such as *P. aerugiosa* PAO1, S. maltophilia K279a, and A. baumannii AYE [41,42,43]. Even in the early days of its development, WGS proved to be a valuable technique capable of providing various genomic features. It revealed not only the numerous genes involved in bacterial regulation, catabolism, transport, efflux, and chemotaxis, but also provided some insights into the adaptive mechanisms and intrinsic drug resistance of some important pathogens. The major barrier to its widespread adoption is hidden in the significant cost of the analysis due to the dye-primer or dye-terminator Sanger sequencing approaches used [44,45]. The development of new sequencing technologies, united under the collective term “next generation sequencing” (NGS), has revolutionized the study of bacterial genomes due to their substantially reduced cost and significantly improved time-efficiency [46]. The genome of *A. baumannii* ATCC 17978 is among the first ones obtained by the NGS approach called high-density pyrosequencing [47]. It generated 22 scaffolds, which ranged in length from 6199 base pairs (bp) to 1,257,593 bp with an average of 179,384 bp. All gaps were subsequently filled by the employment of PCR-based strategies. They added 30,304 bp in total, indicating that the pyrosequencing effectively determined 99.24% of the total chromosome sequence.

The further development of these methods has allowed the use of WGS not only for scientific research purposes, but also in many clinical applications, such as isolate identification, AMR profiling, and outbreak investigation. A good example of such implementation is the foodborne disease surveillance program provided by the global PulseNet laboratory network. The participating laboratories have preferred WGS over pulse-field gel electrophoresis as the primary monitoring tool due to demonstrated superior sensitivity, specificity, and more timely resolution of outbreaks [48]. Sequencing and analyzing the bacterial genomes are becoming increasingly widespread on a global scale and have transformed many of the current protocols in bacterial genetics [49]. The Balkans are not an exception from this trend and a significant number of local reports utilizing NGS for identification, resistome, and virulome analysis, as well as monitoring the emergence and spread of nosocomial pathogens, exist in the literature [50,51,52,53,54,55,56]. Having in mind the clinical significance of the multidrug-resistant NFGNB, such as *A. baumannii*, *P. aerugiosa*, and S. maltophilia isolates, it will be important to summarize the findings of WGS-based resistome analyses and the molecular epidemiology of infections caused by these pathogens in the Balkan countries. In order to better understand these data, one should first focus on the sequencing technologies used that have made it possible to generate large amounts of sequence data rapidly and at a substantially lower cost.

All studies that sequenced genomes of clinical isolates from Balkan countries used some of the existing high throughput DNA sequencing methodologies [57,58]. Second generation methods based on sequencing by synthesis (SBS) are the most preferred choice mainly due to their highest cost-efficiency per analysis among all the existing NGS technologies. These approaches produce millions to billions of short reads with intrinsically higher error rates compared to Sanger sequencing [59]. The genome assembly from these reads can either be performed de novo or by mapping them to a reference strain [60]. The lower accuracy of the SBS dictates that each sequenced bacterial genome has to be generated as a set of consensus sequences that are composed by many overlapping sequencing reads. The low length of the individual sequencing reads affects the assembly level of the de novo sequenced genomes leading to the production of draft versions composed by tens to hundreds of unplaced pieces called contigs [61]. The linear order of such segments cannot be determined by the DNA assemblers, but it may be estimated afterwards via comparison with single complete reference genome using stand-alone software tools like Mauve [62] or by multiple reference-based scaffolders that utilize a set of refence genomes [63,64,65,66]. It is worth noting that this type of in silico contig re-arrangement assumes similarity between the architectures of the sequenced and the reference genomes and, therefore, it may not accurately reveal some isolate-specific genomic rearrangements. In general, it has a limited impact on analyses focused on the resistome composition despite the fact that an antibiotic resistance determinant may be missed when analyzing a de novo assembled genome if it was split across multiple contigs during the assembly procedure [60]. Furthermore, AMR-related mutations affecting only one or two copies of multicopy genes can be omitted when the assemblers collapse these sequences into a single copy. Such cases have been described for genetic variants in the 23S rRNA responsible for macrolide-lincosamide-streptogramin (MLS) resistance in *Neisseria gonorrhoeae* and other microorganisms [67,68,69]. Another shortcoming of the de novo assembled genomes from short sequencing reads affects our ability to properly localize the identified AMR genetic determinants. In such draft sequences, the contig breaks are usually caused by the presence of repeated sequences, such as insertion sequence (IS) elements, transposons, Clustered Regularly Interspaced Short Palindromic Repeats (CRISPR) arrays, rRNA genes, etc. As already mentioned, genome assemblers trend to collapse these regions into a single copy creating contig borders in the corresponding locations. This leads to inability to pinpoint the exact location of many of the identified AMR resistance genes and cassettes that are flanked by ISs and/or are part of structures, such as integrons and transposons. Some of the most troublesome resistance mechanisms in NFGNBs include production of Verona integron-encoded [70,71] and Imipenemase (IMP)-type [72,73] MBLs in *P. aeruginosa* and the acquisition of *sul* genes that are part of 3′ conservative ends of class 1 integrons in *S. maltophilia* [74,75], so this limitation is not insignificant.

On the other hand, the reference-based genome assemblies do not have problems with fragmentation/contig ordering, but they require that a high-quality reference genome is available and the detection of single nucleotide polymorphisms becomes less accurate when the isolate and the reference are not highly similar. This may affect the proper detection of chromosomal mutations with significant contribution to the AMR in NFGNB, such as the ones found in the quinolone resistance-determining regions of *gyrA* and *parC* [76,77,78] or those related to polymyxin resistance in various genes [79,80,81].

Two different types of SBS-based NGS methodologies have mainly been used for sequencing the genomes of NFGNB clinical isolates from the Balkans. These are the Illumina technology that was first developed by Solexa and Lynx Therapeutics [82] and the DNA nanoball approach [83] commercialized by the BGI Group. Comparative analyses demonstrate that platforms based on both approaches produce data with comparable magnitudes of error and can be used interchangeably for genome sequencing [84].

A limited number of studies of NFGNB genomes from the Balkans also utilize long sequencing reads generated by Oxford Nanopore Technologies (ONT) platform. The methodology for DNA sequencing by passing long DNA molecules through small diameter pores and measuring the currents as each nucleotide passes can be considered as a “fourth generation” technology [57]. It is characterized by the production of very long reads (from hundreds of kilobases up to megabases) that can span the repetitive regions in bacterial genomes but have considerably higher error rates compared to the ones generated by SBS methods. These long reads can be used for the generation of high-quality complete circularized bacterial genomes (including the plasmids as separate sequences). The major limitations of the ONT platforms are still the higher cost per genome and the sequence errors that set a challenge for accurate genomic analyses. The most efficient approach to overcome the limitations of both technologies is to use a hybrid assembly strategy that utilizes ONT and SBS sequencing reads together [85].

Knowing the general strengths and the weaknesses of the described second and fourth generation sequencing methodologies, we can now proceed with describing how WGS was applied to study the resistomes of clinical NFGNB isolates from the Balkans.

## 4. *P. aeruginosa*

### 4.1. Mechanisms of Carbapenem Resistance in P. aeruginosa

*P. aeruginosa* has a genome with an average size of 6.7 Mbp, a median number of 6016 coding sequences, and a GC content of 66.1% [86]. Recent analysis of the species pangenome indicated more than 16,000 non-redundant genes and only approximately 15% of them are included in the core genome. This genome variability at the strain level corrupts the efforts to develop a vaccine that can establish complete protection against *P. aeruginosa* infections [87]. Moreover, it also highlights the importance of WGS for proper characterization of isolates and the molecular epidemiological investigation of outbreaks. As mentioned, CRPA isolates represent one of the major hazards in hospital settings impelling a great challenge to the treatment of infected patients. *P. aeruginosa* can develop a carbapenem resistance utilizing various mechanisms. Some of them are driven by acquisition of chromosomal mutations. For example, early reports pointed out genetic variations leading to loss of the outer membrane protein OprD resulting in altered permeability as the predominant cause for a reduced susceptibility to imipenem [88,89]. Later studies revealed that a regulatory gene for *oprD* also contributes to carbapenem resistance [90]. The overexpression of three-component efflux systems, such as MexAB-OprM, MexCD-OprJ, and MexXY-OprM, by mutations at the regulatory regions extrudes a wide variety of antimicrobial agents, including meropenem (but not imipenem) [91,92]. Mutational derepression of the chromosomally-encoded cephalosporinase AmpC in combination with an exchange to alanine at position 105 lead to reduced susceptibility against oxyiminocephalosporins and imipenem [93]. If CRPA isolates possess combinations of chromosomal mutations that lead to OprD loss, MexAB-OprM overexpression, and AmpC derepression, their synergistic impact can confer high-level resistance to carbapenems with minimal inhibitory concentrations (MICs) up to and even higher than 128 mg/L [94].

Despite that the acquisition of chromosomal mutations is the predominant way for *P. aeruginosa* to develop a carbapenem resistance, another mechanism that deserves special attention is the production of carbapenem-hydrolysing enzymes (carbapenemases). They can easily spread between isolates via horizontal gene transfer through integrons, transposons, and plasmids accelerating the dissemination of CRPA isolates [86]. Three molecular classes of carbapenemases, named A, B, and D, have been identified in *P. aeruginosa* so far [71,95].

The class A β-lactamases utilize Ser residue at the active site and an additional Glu residue is involved in the catalytic process [96,97]. Their activity is partially inhibited by clavulanic acid. GES-type extended-spectrum β-lactamases (ESBLs), belonging to class A, have been increasingly reported among Gram-negative pathogens, including *P. aeruginosa* and *A. baumannii*. Some GES-type enzyme variants, including GES-2, 4, 5, 6, and 14, have shown carbapenem-hydrolyzing activity [98,99,100,101,102,103]. GES-5 was reported in a clinical *P. aeruginosa* isolate obtained from a tertiary hospital in Istanbul, Turkey a few years ago [104]. Later, the same enzyme, together with GES-1, was also described in clinical CRPA isolates from Bulgaria [105]. *Klebsiella pneumoniae* carbapenemase (KPC) is another member of class A β-lactamases that has been identified in *Pseudomonas* and *Acinetobacter* spp. In 2007, the first clinical isolate of KPC-producing *P. aeruginosa* was identified in a Colombian hospital [106] and has continuously spread throughout other countries, including the USA, China, Brazil, and Germany [107,108,109,110].

The class B comprises MBLs and is the most prevalent among clinical isolates on a global scale. MBLs can hydrolase all β-lactams except for monobactams, such as aztreonam. Their activity can be inhibited by the presence of a chelator-like ethylenediaminetetraacetic acid (EDTA) because they utilize divalent cations as a cofactor. Clinically relevant small molecule inhibitors that can block the MBL action have not been found yet [111]. Several types of MBLs have been recovered from clinical *P. aeruginosa* isolates. The first IMP-type MBL was recovered from an imipenem-resistant strain *P. aeruginosa* GN17203 in Japan in 1991 [112]. Today, more than 70 IMP variants are known and this MBL is the second most common carbapenemase produced by *P. aeruginosa* [86]. The VIM-1 enzyme was first isolated from a CRPA strain in Verona, Italy [70]. Another subtype named VIM-2 was next obtained from a blood culture of a 39-year-old woman treated with imipenem in Marseilles, France in 1996 [113]. Both these MBL_S_ were originally found in *P. aeruginosa* as parts of gene cassettes in the variable regions of class 1 integrons, and VIM-producing clinical isolates have been detected all over the world. Numerous reports for their identification on the Balkans exist [114,115,116,117,118,119] highlighting them as a serious threat to the public health in this region. The first member of the New Delhi metallo-β-lactamase (NDM)-type carbapenemases was recovered from a Swedish patient who had recently travelled to India prior to hospitalization [120]. Moreover, the majority of the European NDM-1-producing *Enterobacterales* clinical isolates in the next few years were isolated from patients who had recent travel and hospitalization in India, Pakistan or the Balkan countries [121]. The NDM-1 variant was detected for a first time in *P. aeruginosa* on the Balkans in Serbia [122]. CRPA isolates expressing MBLs different from the globally distributed VIM-, IMP-, and NDM-variants do exist, but they have limited significance for the Balkan region since the local screening efforts have not yet detected them [123].

The Class D carbapenemases are serine β-lactamases with a key carboxylated Lys residue that is responsible for the hydrolysis [124]. They are also named oxacillinases (OXAs) in reference to their ability to hydrolyze oxacillin much faster than benzylpenicillin. Twelve groups of these enzymes exist, and three of them (OXA-40-like, OXA-48-like, and OXA-198-like) rarely have been identified in *P. aeruginosa* [86]. They have not been described in CRPA isolates on the Balkans so far. It should be mentioned that Turkey, together with North African and Middle East countries, are among the most important reservoirs for plasmid-borne OXA-48-like variants in *Enterobacterales*, so a realistic possibility for horizontal gene transfer exists [125].

The global surveillance initiatives have identified ten *P. aeruginosa* high-risk clones in terms of prevalence, global spread, and association with MDR/XDR profiles—ST235, ST111, ST233, ST244, ST357, ST308, ST175, ST277, ST654, and ST298 [126]. Two of them, ST235 and ST111, are the carbapenemase producers that raise the biggest concerns since they are associated not only with class B but also with class A and D carbapenemases [86]. Strains that belong to both clones have been identified in many of the Balkan countries. Most of the WGS-subjected clinical CRPA isolates in the region cover members of these two clones.

The studies to date, including WGS-based resistome analyses of problematic *P. aeruginosa* clinical isolates from the Balkan countries, are presented in the next few subsections. Summary data from them are shown in Table 1.

### 4.2. WGS of Clinical P. aeruginosa Isolates in Albania

In their study, Tafaj et al. report the genome sequences of two NDM-1-producing *P. aeruginosa* strains of ST235 that were isolated from the surgical wound of two inpatients in Tirana [127]. The WGS procedure was carried out at the San Raffaele Hospital (Milan, Italy) using Illumina NextSeq 500 (2 × 150 bp) sequencing. It generated two draft genomes, each composed by more than 500 contigs. Both of them were found to harbor *bla*_NDM-1_ genes in combination with a total of 58 other AMR genetic determinants, including genes conferring resistance to β-lactams, aminoglycosides, fluoroquinolones, macrolides, and tetracyclines, through different mechanisms, such as antibiotic efflux (*n* = 37), antibiotic efflux and antibiotic target alteration (*n* = 3), antibiotic inactivation (*n* = 11), antibiotic target alteration (*n* = 6), and antibiotic target replacement (*n* = 1) [127]. All of them were identified using the Resistance Gene Identifier (RGI) v.5.1.0 from the Comprehensive Antibiotic Resistance Database (CARD) [133]. A significant limitation of this study remains the absence of antibiotic susceptibility testing results meaning that the performed resistome analysis is only computational. It remains unclear if all these in silico predictions result in AMR phenotypes.

### 4.3. WGS of Clinical P. aeruginosa Isolates in Bulgaria

In the first report from Bulgaria, Kostyanev et al. described the WGS of five CRPA strains that were obtained from clinical samples of different patients in two hospitals in Sofia [105]. A combination of short-read Illumina MiSeq (2 × 250 bp) sequencing and long-read ONT MinION sequencing was used. The five isolates were found to be clonally related, all belonging to ST654 and all possessing *bla_NDM-1_*MBL genes. This combination of sequencing technologies allowed the hybrid assembly of one chromosome-level genome (GCF_021378395.1). This is the first complete *P. aeruginosa* genome described in a study from the Balkans. Its analysis revealed a novel class 1 integron In1884 with the 5′CS–*bla*_GES-5_/*aadB*–3′CS gene cassette array [105].

The second report analyzed a single XDR *P. aeruginosa* isolate obtained in September 2019 from a urine sample of a 60-year-old male [128]. Authors used the Illumina HiSeq (2 × 150 bp) platform. The assembled draft genome was 7.16 Mb in size, comprising 78 contigs larger than 1000 bp (largest contig—644,223 bp) with an N50 value of 231,855 bp. WGS-based MLST analysis classified the isolate into the globally recognized high-risk sequence type ST111 [134]. The assembled genome was shown to contain a *bla*_VIM-2_ gene as part of the resistance gene cassette embedded into the variable region of its In59-like integron [128].

### 4.4. WGS of Clinical P. aeruginosa Isolates in Greece

A recent large-scale study in Greece screened a total of 120 non-repetitive clinical *P. aeruginosa* isolates, which had meropenem MICs greater than 2 mg/L, for the presence of VIM-genes [129]. Sixty-one CRPAs were found to contain genetic determinants for VIM MBLs and the isolates of ST111 were dominant among them (*n* = 34), followed by those belonging to ST235 (*n* = 15). Next, a PCR-based methodology was used to amplify and sequence the *bla*_VIM_ genes within their corresponding integrons. Six isolates, representative of different integron structures and sequence types (STs), were subjected to WGS using Illumina MiSeq (2 × 300 bp). Each of the resulting genomes was shown to harbor either a *bla*_VIM-2_ or *bla*_VIM-4_ variant. Additionally, the genomes of nine *P. aeruginosa* isolates, being positive in the EDTA–meropenem test but negative in PCR screening, were also sequenced. No known MBL genes were found in them suggesting that the phenotypic detection of MBLs using double-disk synergy test with imipenem-EDTA may be unreliable on some occasions [135].

### 4.5. WGS of Clinical P. aeruginosa Isolates in Romania

In order to estimate the effects of the high consumption of antimicrobials in Romania on the AMR profile of NFGNBs, Gheorghe-Barbu et al. sequenced the genomes of 34 MDR *A. baumannii* and 20 MDR *P. aeruginosa* strains [130]. These isolates were recovered in the 2018–2019 period from hospital settings, hospital collecting sewage tanks, and the receiving wastewater treatment plants located in seven different geographic locations, and the sequencing was performed by Illumina MiSeq (2 × 300 bp). In the context of the present review article, the detection of dissemination of *bla*_IMP-13_ resistance determinants among isolates from a Bucharest hospital and its effluent is very alarming. A major limitation of this study is the way of presenting the results from the resistome analyses. It is focused entirely on summarization of all data into a single table and suffers by the insufficient description of the individual isolates and their AMR determinants [130]. Despite that, it successfully demonstrates how WGS could provide important information about AMR determinants’ transmission from the hospital environment to wastewater.

### 4.6. WGS of Clinical P. aeruginosa Isolates in Serbia

A recent study investigated the molecular characteristics of MBL-producing CRPA isolates in Serbia, as well as the underlying resistance mechanisms and the genetic context of the MBL genes detected [131]. Their distribution suggested clonal dissemination and possible recombination. High-risk clones ST235 and ST654 were identified for the first time in Serbia in combination with *bla*_NDM-1_ determinants that confer resistance to carbapenems and all other β-lactams, except for aztreonam. Kabic et al. also performed detailed phylogenomic analysis by calling and comparing single nucleotide polymorphisms (SNPs) from the core gene alignment of 165 *P. aeruginosa* genomes, of which four were sequenced by the authors. It should be mentioned that this is the second study form the Balkans that utilizes ONT long reads (MinION) in combination with the short reads generated by an Illumina platform (Illumina HiSeq, 2 × 150 bp) [131]. This results in the generation of the second complete genome of a CRPA isolate again carrying the *bla*_NDM-1_ gene similar to the report from Bulgaria [127]. The absence of essential clinical information regarding the isolates that were subject to WGS can be considered as the biggest limitation of this study.

### 4.7. WGS of Clinical P. aeruginosa Isolates in Turkey

Çekin et al. identified two carbapenemase-producing isolates from a Turkish hospital by both Carba NP [136] and Carbapenem Inactivation Method [137] tests and sequenced their genomes using Illumina MiSeq (2 × 150 bp) [132]. In addition to the identified *bla*_VIM-5_ and *bla*_IMP-7_, the genomes of the two CRPA strains also harbor plasmid-borne resistance determinants, such as *crpP* (ciprofloxacin resistance protein, plasmid encoded) and *crpP-2* [138]. The *crpP* gene was also found in the VIM-2 producing isolate previously described in Bulgaria [128].

### 4.8. WGS of Clinical P. aeruginosa Isolates from the Balkans in International Projects

A number of large-scale international projects that use WGS to analyze clinical *P. aeruginosa* isolates exist, and some of them include isolates from Balkan countries [139,140,141,142].

In summary, the main goal of all WGS-based resistome analyses carried out in the Balkan countries in recent years has been to explore the mechanisms of carbapenem resistance, especially MBL genes and their genetic context. A variety of MBL genes have been identified, including *bla*_NDM_, *bla*_VIM_, and *bla*_IMP_ alleles. All investigated *P. aeruginosa* isolates from Albania, Bulgaria, Serbia, and Turkey, as well as most isolates from Greece and Romania, have been classified into globally recognized epidemic high-risk sequence types (STs: 111, 235, 308, 357, and 654) [126]—Table 1.

## 5. *A. baumannii*

### 5.1. Mechanisms of Carbapenem Resistance in A. baumannii

Analysis of all complete *A. baumannii* genomes available at the National Center for Biotechnology Information (NCBI) reveals that the size of species chromosome varies in the range of 3.63–4.57 Mbp with a GC content of 38.76–39.7%. Recent pan-genome analysis of 79 *A. baumannii* genomes identified 1344 core, 4644 accessory, and 1695 unique protein-coding genes [143]. Unique genomic content was presented mainly by genetic determinants that contribute to carbon catabolism, virulence, and antibiotic resistance. The extent of AMR and its environmental flexibility are the two key aspects responsible for the ubiquitous dissemination of *A. baumannii* in hospitals worldwide [144].

Carbapenems have generally been considered as the preferred antibiotics to treat *A. baumannii* infections due to their efficiency and favorable safety [145]. Due to this, CRAB isolates are considered as a major threat in hospital settings and represent a critical challenge to the treatment of infected patients. It is worth noting that the carbapenem resistance is widely spread among invasive *Acinetobacter* spp. isolates on the Balkans.

*A. baumannii* utilizes numerous AMR mechanisms, including β-lactamases/carbapenemases, aminoglycoside-modifying enzymes, overexpression of efflux pumps, permeability defects due to outer-membrane proteins (OMPs) loss, and modifications of target sites [22,145,146,147].

The carbapenemase-mediated resistance in *A. baumannii* needs special attention since the species was shown to possess natural competence to incorporate exogenous DNA [148,149]. The considerable amount of foreign DNA in its genome suggests frequent horizontal gene transfers in this pathogen. Moreover, albumin, the main protein of the blood plasma, enhances the natural competence of *A. baumannii*, which leads to even higher possibilities for carbapenemase gene acquisition in invasive isolates [150]. All three molecular classes of carbapenemases (A, B, and D) have been found in clinical CRAB isolates. Class A β-lactamases detected in *A. baumannii* are represented by GES-11 found in France [151] and KPC-2, 3, 4 and 10 initially detected in Puerto Rico [152] and Brazil [153]. The different KPC variants are usually found in MDR-CRAB isolates, which cause difficult to treat infections with high mortality rates. KPC carbapenemases were also frequently found in CRAB isolates obtained from burn victims [154]. A variety of class B MBLs have been detected in CRAB strains so far, including IMP-1 [155], IMP-2 [156], IMP-4 [157], IMP-5 [158], IMP-6 [159], IMP-8 [160], IMP-11 [161], IMP-19 [161], IMP-24 [160], IMP-55 [162], NDM-1 [163], GIM-like [164], NDM-2 [165], NDM-3 [166], SIM-1 [167], VIM-1 [168], VIM-2 [169], VIM-3 [160], VIM-4 [170], and VIM-11 [160]. Although MBLs are not the predominant type of carbapenemases found in *A. baumannii*, they present a serious threat for infected patients due to their broad substrate range, potent activity, and resistance to all available inhibitors [144]. The most important class of carbapenemases in clinical CRAB isolates are the OXA-type Class D β-lactamases [22]. So far, more than 400 OXA-enzymes have been identified among various bacteria [146] and many of them possess carbapenem hydrolyzing activity [171]. The corresponding *bla*_oxa_ genes can be located either on the chromosome, on a plasmid, or sometimes may be found in integrons. Carbapenem-hydrolyzing class D beta-lactamases (CHDLs) are the major cause for carbapenem resistance in *A. baumannii* and the acquisition of their corresponding genes is often mediated by flanking IS elements [172]. Four OXA-type CHDLs groups, such as OXA-23, OXA-40/24, OXA-51, and OXA-58, are the predominant carbapenemases in CRAB isolates [144]. OXA-23 was the first one found in *A. baumannii* isolate obtained from the blood culture of a Scottish patient in 1985 [173]. Currently, it is disseminated worldwide, including on the Balkans [174]. The OXA-40/OXA-24 group includes the enzymes OXA-25, OXA-26, OXA-40, and OXA-72 that differ only in few amino acids [175]. From them, OXA-72 has been identified as a cause for an *A. baumannii* outbreak in Croatia [176]. OXA-51-group carbapenemases are intrinsic chromosomal enzymes found in the genome of the species that are expressed at a low level [177]. The acquisition of a strong promoter by insertion of the IS*Aba1* element upstream of the 5′ end of the OXA-51-group gene leads to elevation in the enzymatic activity [178]. OXA-58 is encoded by the *bla*_OXA-58_ gene, which was found to be plasmid borne. A number of outbreaks caused by this variant have been reported in many countries, including Greece [179] and Turkey [180]. Again, OXA-58 can mediate high-level carbapenem resistance in *A. baumannii* either by an upstream insertion of the IS*1008* element [181] or by the presence of the IS*Aba825*-IS*Aba3*-like hybrid promoter [182]. The other three OXA-type CHDL groups found in CRAB, OXA-149, OXA-182, and OXA-235 are rare [183,184,185] and have so far not been identified in *A. baumannii* isolates from Balkan countries.

Multidrug efflux systems can also play a role in the carbapenem resistance of CRAB isolates. In a recent study, high expression of the RND-type pump AdeABC was associated with meropenem resistance [186]. AbeM, an H^+^-coupled Multidrug and Toxic compound Extrusion (MATE) family pump, was reported to confer an imipenem resistance in *A. baumannii* [187].

Permeability defects caused by mutations also contribute to the carbapenem resistance in *A. baumannii*. The loss of the 29 kDa outer membrane porin CarO leads to both imipenem and meropenem resistance [188]. Other studies have also highlighted 22–33-kDa OMP [189], 33–36-kDa OMP [190], 37-kDa OMP [191], 44-kDa OMP [191], and 47-kDa OMP [191] as being involved in the carbapenem resistance.

The alteration of target sites in rare cases can lead to imipenem resistance in the absence of other known resistance mechanisms. This phenomenon was observed in *A. baumannii* isolates that demonstrate overexpression of certain penicillin-binding proteins with a low affinity for imipenem [192].

### 5.2. Mechanisms of Colistin Resistance in A. baumannii

The high rate of carbapenem resistance among the clinical *A. baumannii* isolates on the Balkans forces the clinicians to look for alternative antibiotics to treat the infected patients. Unfortunately, very frequently the CRAB isolates turn to be also MDR or XDR, which severely limits the available treatment options. In such cases, the polymyxin antibiotic colistin frequently is being applied as a “last resort” antibiotic despite its strong neuro- and nephrotoxicity. However, since 2015, its efficiency has been largely compromised by the emergence and rapid dissemination of mobile colistin resistance (*mcr*) genes among Gram-negative bacteria worldwide. Until now, ten MCR-family genes and their variants have been described [193,194]. Despite that *mcr* genes have already been found on the Balkans [195,196,197,198], none of them were obtained from *A. baumannii* isolates. Recent studies from Croatia detected colistin resistant *A. baumannii* isolates, but the resistance mechanism was mediated by chromosomal mutations [199,200]. Nevertheless, these findings are very troublesome especially in the light that some of the strains were also carbapenem-resistant [200].

The chromosomally mediated colistin resistance can occur in *A. bumannii* by several mechanisms [201,202]. The first relies on the complete loss of lipopolysaccharide (LPS) production by mutational inactivation of a lipid A biosynthesis gene (*lpxA*, *lpxC*, or *lpxD*) or via insertional inactivation of *lpxACD* genes due to the IS*Aba11* element [203]. Another way is based on the occurrence of point mutations in *pmrA* and *pmrB* genes of the PmrAB two-component system [203]. Such sequence variations in *pmrB* were found in multiple *A. baumanii* isolates from the Balkans. In addition, a mutation in the *pmrC* homologue *eptA* and a point mutation in IS*Aba1* upstream of *eptA* recently were associated with colistin resistance and increased *eptA* expression [204].

The studies to date, including WGS-based resistome analyses of problematic *A. baumannii* clinical isolates from the Balkan countries, are presented in the next few subsections. Summary data from them are shown in Table 2.

### 5.3. WGS of Clinical A. baumannii Isolates in Albania

In their study, Abdelbary et al. presented the genome sequences of two clinical CRAB isolates obtained from Albanian and Togolese patients [205]. The draft genome sequence of the Albanian isolate was assembled using reads from Illumina MiSeq platform (2 × 150 bp). It was composed by 128 contigs that comprised 3,933,485 bp with an N_50_ contig size of 125,943 bp and a GC content of 38.8%. No antibiotic susceptibility testing results were given, so the resistome analysis was performed entirely in silico.

### 5.4. WGS of Clinical A. baumannii Isolates in Croatia

Seven colistin-resistant *Enterobacterales* and three colistin-resistant CRAB isolates were subjected to WGS in a recent study [200]. It also utilized different NGS technology in the face of the Ion Torrent PGM platform (400 bp). D’Onofrio et al. identified missense mutations in the *pmrB* gene that can be a plausible explanation for the observed colistin resistance. It is worth noting that one of these variants (A138T) was present in all three genomes investigated. In addition, the authors provided complete clinical details about all isolates. The most important conclusion from this work is that colistin-resistant and CRAB isolates have already emerged on the Balkans and the clinicians in local hospitals should be prepared to apply novel combined strategies in the treatment of severe infections caused by colistin-resistant CRAB isolates [212].

### 5.5. WGS of Clinical A. baumannii Isolates in Greece

The first report from Greece described the complete genome of the *A. baumannii* isolate A388 recovered in 2002 [206]. It represented a distinct antibiotic-resistant lineage of the global clone 1 (GC1) producing OXA-58 carbapenemase. Authors used long reads generated by the MinION platform (ONT) to generate the 4.332-Mbp genome sequence. Curiously, the short reads used for the hybrid assembly were generated long ago in a previous study, using the llumina HiSeq platform (SRA accession number ERX087515). Taking this into consideration, the work of *Hamidian* et.al. demonstrates that the portable sequencing device MinION can be used to complete previously sequenced genomes.

The second Greek study concentrated on genome analysis (Illumina NovaSeq, 2 × 150 bp) of 40 colistin-resistant CRAB isolates. Two genomes of colistin-susceptible *A. baumannii* were also sequenced for comparison [207]. It is worth noting that the isolates, analyzed by Palmieri et al. were isolated before the colistin-resistant CRAB strains from Croatia. Authors identified a previously described mutation in *pmrB* (A226V) in all resistant isolates. It was associated with low-level colistin resistance before [79,213]. Some genomes harbored additional mutations in *pmrB* (E140V or L178F) or *pmrA* (K172I or D10N), first described by the authors. They resulted in higher colistin MICs for the corresponding isolates. In addition, the A138T mutation (found also by D’Onofrio et al.) was observed in all genomes sequenced suggesting that it has no role in the colistin resistance phenotype as previously reported [214]. Finally, all isolates were found to harbor mutations in the QRDR regions of *gyrA* and *parC* that confer quinolone resistance.

### 5.6. WGS of Clinical A. baumannii Isolates in Romania

Gheorghe et al. presented their report on the resistome and virulome of seven XDR/CRAB strains isolated from hospitalized and ambulatory patients in Bucharest, Romania [208]. The analysis revealed AMR genetic determinants that are present in all strains as well as some resistance genes that are isolate-specific. The entire study is well designed, and the authors provide very detailed clinical information for all isolates tested. All data from the assembly of the genomes (performed using paired-end reads by Illumina HiSeq and MiSeq platforms) and the corresponding resistomes are given.

### 5.7. WGS of Clinical A. baumannii Isolates in Serbia

The first report from Serbia presented the draft genome sequence (Illumina MiSeq 2 × 75 bp) of a clinical CRAB isolate [209]. Authors did not provide any further details. The isolate was found to harbor the *bla*_OXA-72_ carbapenemase gene for a first time in Serbia. Its draft genome sequence consisted of a 3.91 Mbp, with an average GC content of 38.8%.

The second report described the WGS (Illumina MiSeq) of 30 colistin-resistant *A. baumannii* isolates and analyzed the global genomic epidemiology of these infectious agents [210]. Phylogenomic analysis showed that colistin resistance arose independently in several clonal lineages. Mutations in the PmrB and subsequent overexpression of the phosphoethanolamine transferase PmrC were found to be the major mechanism of colistin resistance among the tested isolates. Also, one of the colistin-resistant isolates was found also to possess the *bla*_NDM-1_ gene. The presence of MBL in an isolate that is not susceptible to colistin is alarming since such combined resistance mechanisms will create extreme difficulties to the local clinicians on the Balkans.

### 5.8. WGS of Clinical A. baumannii Isolates in Turkey

In their study, *Gülbüz and Sariyer* analyzed a MDR *A. baumannii* strain via WGS (Illumina NovaSeq) [211]. In addition to the standard resistome and virulome analyses, authors also performed homology modelling, molecular docking, and dynamics simulations in order to obtain complete structural information about the G225S mutation found in the *bla*_ADC-73_ β-lactamase of the isolate.

### 5.9. WGS of Clinical A. baumannii Isolates from the Balkans in International Projects

Large scale WGS of CRAB isolates was performed within the EURECA study [215]. In total, 228 CRAB strains from 10 countries were collected from blood cultures and their corresponding genome assemblies were obtained via Illumina sequencing. The majority of the isolates originated from patients hospitalized in Balkan countries, predominantly Serbia (*n* = 105), Greece (*n* = 41), and Kosovo (*n* = 32).

In summary, the main goal of the WGS-based resistome analyses regarding problematic nosocomial *A. baumannii* isolates from the Balkans has been to explore the mechanisms of carbapenem resistance, as well as colistin resistance. A variety of CHDL genes and ISs have been identified, while genes for MBLs are rarely found. Also, pandrug-resistant *A. baumannii* isolates have already emerged in Croatia.

## 6. *S. maltophilia*

### 6.1. Mechanisms of Antibiotic Resistance in S. maltophilia

The clinical *S. maltophilia* K279a isolate, obtained from a blood sample of an elderly male patient undergoing chemotherapy, was shown to possess a genome with a total size of 4,851,126 bp and a GC content of 66.7% [42]. Its analysis indicated the presence of nine RND-type efflux pump genetic determinants identified on a sequence homology basis. Gene disruption experiments demonstrated their involvement in the intrinsic drug resistance of the isolate by decreasing the MICs of aminoglycosides, fluoroquinolones, and tetracyclines but none dramatically [42]. It is known than the MDR phenotype in Gram-negative nosocomial pathogens is frequently mediated by the over-expression of such RND-type efflux pumps [216,217]. With the development of the NGS technologies that lead to wider adoption of WGS in the bacterial genomics, more *S. maltophilia* genomes become available [218]. Their analyses revealed that the species possesses a variety of AMR genetic determinants, including β-lactamases and aminoglycoside modifying enzymes, in addition to the efflux pumps [31]. Importantly, these genes were found in most of the analyzed isolates, showing the same synteny and high levels of sequence homology. This observation suggests that such AMR genetic determinants have not been transmitted recently in *S. maltophilia* but are rather old and were acquired before the antibiotic therapy was introduced. Moreover, some of these genes have important functions in *S. maltophilia* physiology, such as the genes encoding the SmeDEF efflux pump. Its activity is essential for the colonization of plants roots [219] and at the same time it is involved in resistance to quinolones, tetracyclines, macrolides, chloramphenicol, and novobiocin [220]. The ambivalent nature of the AMR determinants to a large extent explains the intrinsic low susceptibility of *S. maltophilia* to most of the antibiotics allowing this opportunistic pathogen to infect patients receiving antimicrobial therapy. Actually, previous antibiotic treatment can be considered even as a risk factor for such infection [221,222]. This hypothesis is supported by the observation that *S. maltophilia* is the most common pathogen detected in the severe group and in the critical group of COVID-19 patients in the ICU at the Beijing Ditan Hospital in Beijing, China [29].

*S. maltophilia* has an intrinsic resistance against β-lactam antibiotics (including carbapenems). Genome analyses revealed that it possesses two chromosomally encoded β-lactamases named L1 and L2. The L1 enzyme was classified as a Zn^2+^-dependent class B3 MBL [223], while L2 is a serine active-site class A cephalosporinase [224] susceptible to clavulanic acid. When a β-lactam antibiotic is present, the expression of both enzymes gets stimulated via AmpR-dependent upregulation [225]. In addition, *ampD(I)* [226] and the *ampN*-*ampG* operon [227] also are essential for the β-lactamase inducibility.

Besides β-lactamase genes, the chromosome of *S. maltophilia* harbours genes for several intrinsic aminoglycoside-modifying enzymes, including *aac(6′)-Iz* (encodes aminoglycoside acetyltransferase that confers resistance to amikacin, tobramycin, sisomicin, and netilmicin) [228], *aph(3′)-IIc* (encodes aminoglycoside phosphotransferase that contributes to resistance to kanamycin, neomycin, paromycin, and butirosin) [229], and *aac(6′)-Iak* (encodes aminoglycoside acetyltransferase that decreases the susceptibility to arbekacin, kanamycin, neomycin, sisomicin, and tobramycin) [230].

The major contributors to the intrinsically high antibiotic resistance of *S. maltophilia* are the various types of efflux pumps, which can be found encoded in its genome. They include: two ATP-binding cassette (ABC) multidrug efflux pumps—SmrA (contributes to fluoroquinolones, tetracycline, and doxorubicin resistance) [231] and MacABCsm (contributes to aminoglycosides, macrolides, and polymyxins resistance) [232]; one major facilitator superfamily (MFS) efflux pump EmrCABsm (participating in the export of nalidixic acid, erythromycin, carbonyl cyanide 3-chlorophenylhydrazone, and tetrachlorosalicylanilide) [233]; and the fusaric acid tripartite efflux pump FusA (involved in the efflux of fusaric acid) [234]. Furthermore, eight types of RND efflux pumps (SmeABC, SmeDEF, SmeGH, SmeIJK, SmeMN, SmeOP, SmeVWX, and SmeYZ) were also found in the genome of *S. maltophilia*, and studies have identified a role in the antibiotic resistance for seven of them (all except SmeMN) [218]. Some RND pumps have a basal expression level under regular growth conditions that is sufficient to alter the susceptibility to antimicrobials of the bacterium. SmeDEF is the best studied system of this type. Its mutational inactivation leads to increased susceptibility toward several antimicrobials, including quinolones, chloramphenicol, tetracycline, macrolides, sulfamethoxazole, trimethoprim, and trimethoprim–sulfamethoxazole (SXT) [220,235,236]. The expression levels of other pumps are increased upon induction by different signals.

Another chromosomal gene that is related to the AMR in *S. maltophilia* is Sm*qnr* [237]. It encodes for a pentapeptide repeat protein that protects the DNA topoisomerases from the action of fluoroquinolones. A large number of Sm*qnr* alleles exist in clinical isolates, each of them presenting subtle differences in its contribution to the quinolone resistance [238].

The extreme variety of intrinsic chromosomally encoded AMR genetic determinants in *S. maltophilia* complicates all attempts for resistome analysis. In principle, the WGS can provide detailed information about sequence variations in the regulatory regions and the coding sequences of all these genes, but the interpretation of the results is difficult and with a low level of confidence. The identified missense mutations are often strain-specific and/or found in unique combinations, which decreases the possibilities to predict their functional outcome. Moreover, the number of sequenced *S. maltophilia* genomes is significantly lower compared to these of *P. aeruginosa* and *A. baumannii*, and this reduces the predictive power of WGS in the species. Additional techniques, such as real-time PCR, transcriptome sequencing, and genetic manipulations, can help to overcome these issues, but most researchers choose not to apply them due to time- or cost-related limitations.

In addition to the intrinsic resistance mechanisms and the acquired antimicrobial resistance via mutations leading to overexpression of pumps, *S. maltophilia* can obtain AMR genetic determinants through horizontal gene transfer of various genetic structures. SXT is traditionally recommended as the first option against *S. maltophilia* infection; however, increasing resistance to this antimicrobial agent has complicated the treatment [239]. Resistance determinants, such as *sul* (encoding dihydropteroate synthases) and *dfrA* (encoding dihydrofolate reductases) genes, class 1–3 integrons, and mobile genetic elements, contribute to SXT resistance [240,241]. Studies have demonstrated that *sul1* (and to a lesser extent *sul2* and *sul3*, which are part of the class 2 and class 3 integrons, respectively) are the leading causes for SXT resistance [75,242]. Moreover, it has been reported that the *dfrA* genes, located in the gene cassettes of the class 1 integrons, lead to a high-level resistance to SXT [240]. Having this in mind, it is clear that the first goal of a resistome analysis in clinical *S. maltophilia* isolates is always to check for the presence of these genes.

### 6.2. WGS of Clinical S. maltophilia Isolates in Bulgaria

The only studies that have sequenced genomes of clinical *S. maltophilia* isolates on the Balkans were performed by Strateva et al. in Bulgaria. In the first of their reports, authors analyzed the resistome of an XDR *S. maltophilia* isolate (SM130 resistant to SXT, levofloxacin, ceftazidime, chloramphenicol, and colistin) that was obtained in 2015 from a tracheobronchial aspirate of a 44-year-old inpatient with clinical symptoms of ventilator-associated pneumonia [243]. The WGS was carried out on an Illumina HiSeq system (Illumina Inc., San Diego, CA, USA) using 2 × 150-bp paired-end sequencing (BGI Group, Hong Kong, China) to generate a genome assembly at the contig level. The isolate was found to harbor a class 1 integron (with *sul1* gene located at its 3′ conservative end) containing resistance gene cassette embedded into the variable region of the integron. It had a length of approximately 3.2 kb and contained the following genes: *bla*_OXA-74_ (encoding an OXA-10 family class D β-lactamase OXA-74), *aac(6′)-Ib-cr* (fluoroquinolone-acetylating aminoglycoside acetyltransferase) and *cmlA7* (chloramphenicol acetyltransferase). Further searching by the authors found the same cassette solely in the variable region of a class 1 integron in a *P. aeruginosa* isolate (EU161636.1) from Budapest (Hungary) [244].

**Figure 3:**
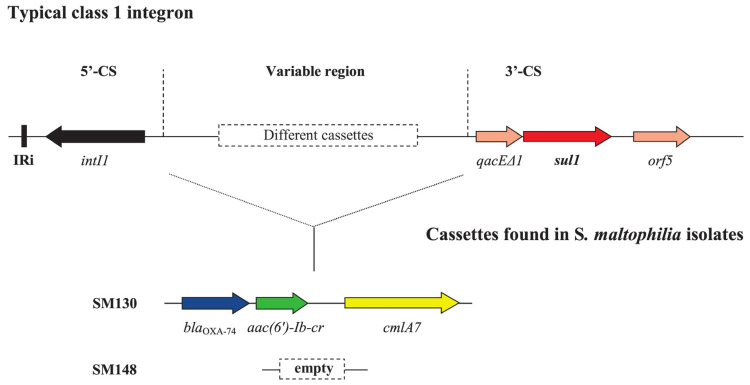
Linear map of a typical class 1 integron. IRi marks an inverted repeat that flanks the *intI1* gene encoding class 1 integrase. The dotted rectangle represents a cassette array with a variable composition. The conserved sequence in the 3′ end of the integron includes *qacEΔ1* (quaternary ammonium compound efflux SMR transporter), *sul1* (dihydropteroate synthase type-1), and *orf5* (unknown function). Below are the two gene cassettes found in isolates SM130 and SM148. The SM130 gene cassette contains the following genes: *bla*_OXA-74_ (OXA-10 family class D b-lactamase OXA-74), *aac(6′)-Ib-cr* (fluoroquinolone-acetylating aminoglycoside acetyltransferase), and *cmlA7* (chloramphenicol acetyltransferase). OXA, oxacillinase; SMR, small multidrug resistance.

Recently, Strateva et al. sequenced eight additional contig-level genomes of nosocomial *S. maltophilia* isolates using the DNA nanoball technology commercialized by BGI Genomics [245]. One of them (SM148) was found to harbor an empty 2.6-kb sized class 1 integron with *sul1* gene corresponding to SXT resistance of the isolate studied. The structure of a typical class 1 integron, as well as that of those recently found in nosocomial isolates from Bulgaria, is presented in Figure 3.

The most important aspect of these Bulgarian investigations was imbedded in the presented data about the strong biofilm forming ability of all isolates. Bacterial biofilms are associated with a variety of infections caused by NFGNB, from those related to medical devices, such as catheters or prosthetic joints, to chronic tissue infections, such as pulmonary diseases of CF patients [246,247]. It is worth noting that bacteria inside a biofilm are much more resistant to antimicrobial agents than planktonic forms. Understanding the interplay between phenotypic and genetic resistance mechanisms acting on biofilms is essential for a complete resistome analysis of biofilm-producing clinical NFGNB isolates.

## 7. Conclusions

The present review shows that XDR and even pandrug-resistant NFGNB strains have already emerged in the Balkan states in recent years. Severe infections caused by these problematic pathogens pose a growing clinical threat to public health; therefore, the development of new antimicrobial strategies should be the future mainstay of infection control stewardship practices in hospitals. Using WGS for AMR monitoring is a superior approach compared to other molecular techniques since it provides a deeper understanding of the genetic resistance mechanisms, as well as pathogen evolution and population dynamics. The resistome analysis can be especially efficient to detect chromosomal mutations, such as those involved in the colistin resistance among NFGNBs.

## Figures and Tables

**Table 1:** Whole-genome sequencing-based resistome studies of carbapenem-resistant, MDR, and XDR *P. aeruginosa* isolates from the Balkans.

Country	Isolates Analyzed	Year	β-Lactam Resistance	Aminoglycoside Resistance	Fluoroquinolone Resistance	Other AMR Determinants	Source
Albania	2 isolates, CRPA, **ST235**	2018	*bla*_OXA-488_, *bla*_NDM-1_, *bla*_PDC-2_	*aac(6′)-Il*, *ant(2*″*)-Ia*, *aph(3′)-IIb*	*cpxR*, *pmpM*, *gyrA* mutations	*bcr1*, *catB7*, *emrE*, *fosA*, *sul1*	[127]
Bulgaria	5 isolates, XDR, **ST654**	2017–2018	*bla*_NDM-1_, *bla*_GES-1_, *bla*_GES-5_	*strA*, *strB*, *aph(3′)-Via*, *aadB*		*sul1*, *sul3*, *tetA*, *tetR*	[105]
	1 isolate, XDR, **ST111**	2019	*bla*_VIM-2_, *bla*_PAO_, *bla*_OXA-395_	*aac(6′)-29a*, *aph(30)-IIb*, *ant(3*″*)-Ia*	*crpP*, *gyrA* and *parC* mutations	*catB7*, *cmlB1*, *fosA*, *sul1*	[128]
Greece	15 isolates, CRPA, **ST111 (x2)**, **ST235 (x6)**, ST162 (x5), ST395 (x2)	2018	*bla*_VIM-2_, *bla*_VIM-4_, *bla*_PAO_, *bla*_OXA-35_, *bla*_OXA-50_, *bla*_OXA-395_, *bla*_OXA-488_, *bla*_OXA-494_, *bla*_PER-1_	*aph(3′)-IIb*, *ant*(2″)*-Ia*, *aadA6*, *aph(3′)-Via*, *aacA4*, *aadA2*, *aacA29*, *strA*, *strB*		*catB7*, *sul1*	[129]
Romania	10 isolates, **ST357**, ST395, ST621	2018	*bla*_IMP-13_	*aph(3′)-IIb*, *ant(2″)-I*		*bcr1*, *catB7*, *fosA*, *sul1*	[130]
Serbia	4 isolates, CRPA, **ST235 (x3)**, **ST654 (x1)**	2018–2021	*bla*_NDM-1_	*aac(6′)Ii*, *aph(3′)-IIb*, *aph(6′)Ib*, *aph(6′)Id*, *aphA6*, *aadA6*		*sul1*	[131]
Turkey	2 isolates, MDR, **ST308 (x1)**, **ST357 (x1)**	2015–2016	*bla*_VIM-5_, *bla_IMP-7_*, *bla*_PAO_, *bla*_OXA-2_, *bla*_OXA-50_, *bla*_OXA-488_	*aac(6′)-1Ib-cr*, *aph(3′)-IIb*, *aac(6′)-Ib3*, *aph(3*″*)-Ib*, *aph(6)-Id*, *aac(6′)-II*, *aadA1*	*crpP*, *crpP-2*	*catB7*, *fosA*, *sul1*	[132]

AMR, antimicrobial resistance; ST, sequence type; NDM, New Delhi metallo-β-lactamase; VIM, Verona integron-encoded metallo-β-lactamase; IMP, Imipenemase-type metallo-β-lactamase; CRPA, carbapenem-resistant *P. aeruginosa*; XDR, extensively drug-resistant. Note: The sequence types given in **red** belong to the worldwide top 10 *P. aeruginosa* epidemic high-risk clones [126].

**Table 2:** Whole-genome sequencing-based resistome studies of MDR, XDR, PDR, and colistin-resistant *A. baumannii* isolates from the Balkans.

Country	Isolates Analyzed	Year	β-Lactam Resistance	Aminoglycoside Resistance	Colistin Resistance	Other AMR Determinants	Source
Albania	1 isolate, CRAB, ST2/ST436	2015	*ampC*, *bla*_OXA-23_, *bla*_MBL_, *bla*_OXA-51_, *bla*_TEM-1_	*armA*, *aph(3′)-Ia*, *aphA6*, *strA*, and *strB*		*sul2*, *tetB*	[205]
Croatia	3 isolates, PDR	2018	*bla*_OXA-23_, *bla*_ADC-25_, *bla*_OXA-66_	*aac(3)-Ia*, *aph(3′)-Via*, *aph(3″)-Ib*, *aph(3′)-Via*, *aph(6)-Id*, *armA*, *aadA1*	*pmrB* mutations: S14L, A138T, S183F, T269P	*catA1*, *sul1*, *tet(B)*	[200]
Greece	1 isolate, CRAB, ST1/ST439	2002	*bla*_OXA-58_	*aphA6*, *aacA4*, *aacC1*, *aphA1*		*sul1*, *tetA*	[206]
	42 isolates, (40 x ColR-CRAB, 2 x CRAB)	2015–2017	*bla*_ADC_, *bla*_OXA–51_, * *bla*_OXA–23_		Several chromosomal mutations in genes potentially involved in colistin resistance	QRDR mutations: GyrA S83L and ParC S80L	[207]
Romania	7 isolates, XDR, ST3636/- (x1), ST492/- (x2), ST1/- (x1), ST636/- (x1), ST2/- (x2)	2017	*bla*_OXA-24_, *bla*_OXA-23_, * *bla*_OXA-23_, *bla*_OXA-51_,* *bla*_OXA-51_, *bla*_OXA-72_, *bla*_ADC-11_, *bla*_ADC-25_, *bla_ADC-30_*, *bla*_ADC-74_, *bla*_TEM-12_, *bla*_TEM-84_, *bla*_PER-1_	*aac(3)-Ia*, *aph(6)-Id*, *ant(3″)-IIa*, *aph(3″)-Ib*, *aadA1*, *aph(3′)-Ia*, *aadA2*, *armA*, *aph(3′)-VIa*, *aph(3′)VIb*, *armA*		*catA1*, *dfrA12*, *msr(E)*, *mph(E)*, *sul1*, *sul2*, *tet(A)*, *tet(B)*, *tetR* QRDR mutations: GyrA S83L and ParC S84L, S467G	[208]
	21 isolates, no antimicrobial susceptibility and ST affiliation data	2018–2019	*bla*_OXA-23_, *bla*_OXA-24_, *bla*_TEM_, *bla*_VIM_, *bla*_VEB_	*aph(6)-Id*, *aph(3′)-Via*, *ant(2″)-Ia*, *ant(3″)-IIa*, *armA*, *aadA1*		*msr(E)*, *mph(E)*, *sul1*, *sul2*, *tet(B)*	[130]
Serbia	1 isolate, CRAB		*bla*_OXA-72_				[209]
	30 isolates, ColR-CRAB	2018–2021	*bla*_NDM-1_, *bla*_OXA-23_, *bla*_OXA-24_, bla*_ADC-30_*, bla*_ADC-73_*, bla*_ADC-74_*, bla*_ADC-217_*	*aadA2*, *aph(3′)-VI*, *aac(3)-Ia*, *aadA*, *aph(3″)-Ib*, *aph(3′)-Ia*, *armA*, *ant(3″)-IIc*, *aph(3′)-Via*, *aph(6)-Id*	Various mutations	*catI*, *dfrA1*, *dfrA12*, *msr(E)*, *mph(E)*, *sul1*, *sul2*, *tet(B)*, *tetR* QRDR mutations: GyrA S84L, V104I, D105E and ParC S81L	[210]
Turkey	1 isolate, MDR, ST218		*bla*_ADC-73_				[211]

AMR, antimicrobial resistance; CRAB, carbapenem-resistant A. baumannii; MDR, multidrug-resistant; XDR, extensively drug-resistant; PDR, pandrug-resistant; ColR, colistin-resistant; ST, sequence type determined using Pasteur Institute typing scheme/Oxford MLST scheme (left/right); MBL, metallo-β-lactamase; QRDR, Quinolone Resistance-Determining Region. Note: * indicates the ISAba1 insertion upstream of the gene encoding respective carbapenemase.

## Data Availability

No new data were created or analyzed in this study. Data sharing is not applicable to this article.
